# Supplementary material for: Are carbon emissions trading and green financial instruments synergistic? -Comprehensive quantitative research based on content analysis
Source: PLoS One. 2024 Mar 7;19(3):e0298601. doi: 10.1371/journal.pone.0298601 (PMC10919601; doi:10.1371/journal.pone.0298601)
Supplement: S1 Data — (ZIP) [file pone.0298601.s001.zip › Data/地级市Z_F工作报告.zip!/╝¬┴╓/╦─╞╜╩╨/└┤╫╘╣1⁄2╓┌║┼í╛┬φ┐╦╩2╛▌═°í┐_2017.docx]

各位代表:

　　现在，我代表四平市人民政府，向大会报告政府工作，请予审议，并请市政协委员提出意见。

　　一、过去四年回顾

　　2013年以来，在省委、省政府和市委坚强领导下，在市人大、市政协监督支持下，市政府紧紧依靠全市人民，坚持“五路并进”，推进“五市联动”，实施“五城联创”，全力建设“美丽四平、幸福家园”，圆满完成本届政府各项目标任务。2016年，地区生产总值预计实现1260.9亿元。地方级财政收入实现63.4亿元。固定资产投资完成893.5亿元，年均增长11%。社会消费品零售总额完成604亿元，年均增长11.2%。

　　——综合经济实力稳步提升。项目支撑引领更加有力。烟厂30万大箱易地改造、君乐宝酸奶乳酸菌等重大项目相继建成投产，共建设亿元以上项目409个。依托东博会、吉商大会等平台广泛开展经贸交流活动，纳入省招商引资调度系统项目454个，到位资金738.2亿元。长平高速改造、四梨大街等重点道路建成通车。以全国第三名优异成绩成为管廊建设国家级试点城市，获得奖补资金10.8亿元。发展内生动力持续增强。首届中国·四平科技成果展洽会成功举办，5个科技成果转化中心落户我市;“黑土地论坛”叫响四平现代农业品牌；负压蒸汽冷凝器等产品填补国内空白。艾斯克等3户企业成功登陆“新三板”。中国驰名商标、吉林省名牌产品分别达到14件、77个。出台工业保增长促转型21条和扶持民营经济发展等惠企扶企政策。2016年民营经济主营业务收入预计实现2884.7亿元，年均增长13.5%。要素保障作用充分释放。市本级财政共建立各类引导资金3.2亿元。供地651.5公顷，供地率达72.4%。深入开展“院士专家四平行”等活动，建立4个院士工作站、6个科技领军人才创业基地、3个博士后工作站，从985、211院校引进硕博人才300余名。制约发展瓶颈有效破解。“两区一城”整合提升，四梨同城化深入推进，辽河垦区升级为省级开发区，新型工业化经济开发区正式获批，二龙湖生态保护示范区积极推进。组建城投公司，与国开行、农发行、交行、建行、浦发行等金融机构广泛开展合作，累计发行地方政府债券32.6亿元，融资106亿元。彻底解决117户企业产权证照等历史遗留问题。

　　——经济结构调整取得进展。“三去一降一补”任务稳步落地。积极化解钢铁、水泥等落后产能，消化房地产库存21.4万平方米、粮食库存30亿斤。定向发行置换债券18.39亿元。扩大企业“助保贷”融资规模，下调失业工伤生育等保险费率，落实用电补贴政策。推进服务业跃升攻坚和城市基础设施重点工程，振兴发展基础不断夯实。工业转型升级步伐加快。累计实施技改项目944项，完成投资1184亿元。嫁接改造效应明显，中航工业、中建材等战略投资者落户四平。省级企业技术中心从29家发展到62家，工业首次投产新产品达到736个，家禽自动掏膛机等新产品实现规模化生产。农机产业链条初步形成,清淤车、翼展车、房车等特种车抢占市场高端，医药产业由传统中药加工向医药健康方向转变，化工园区通过省规划核准，北方光电检测中心即将通过省级认证。创建装备制造国家级产业示范基地和全国质量强市示范城市，成为换热设备产业集群区域品牌示范市。现代服务业发展快速跃升。2016年，服务业增加值预计完成434.6亿元，年均增长7.5％。浪潮四平云计算中心·大数据交易所挂牌成立，四平市电子商务产业园区被确定为“国家电子商务示范基地”。万达城市综合体、宝泰黄金珠宝城等项目投入使用。首届中国·四平满族文化论坛成功举办，北方巴厘岛等景区投入运营。2016年全市接待游客349.2万人次，预计实现收入49.7亿元，创历史新高。现代农业建设成效显著。耕地保护切实加强，建设高标准基本农田63万亩。粮食产量稳定在100亿斤以上，实现“十三连丰”。调减玉米种植面积45万亩，牧业小区发展到1556个，特色棚室蔬菜园区发展到65个。中国·四平农业生产全程机械化峰会成功举办，全市农业耕种收全程机械化水平达到82%。新注册地理标志证明商标4件，“三品一标”农产品达到374个，居全省首位。吉春制药被评为农业产业化国家级重点龙头企业，全市农产品加工业产值预计实现440亿元。农村土地确权全面完成实测任务。累计发放土地收益保证贷款8亿元。农民合作社和家庭农场分别达到7718个和2280家。

　　——城乡建管能力切实加强。规划水平不断提升。编制完成城市总规、四梨同城化、地下综合管廊、海绵城市、棚户区改造、城市供水工程等重大规划，城市发展的系统性、整体性明显增强。基础设施建设强力推进。城市规划馆、旭日立交桥成为地标性建筑，紫气大路、东丰路公铁立交桥开工建设。地下综合管廊形成廊体23公里，海绵城市建设成为全省试点，中心城区供热、燃气、排水管网基本实现全覆盖。市政道路总长度达到422公里，全市公路总里程达到9992公里。城乡面貌大幅改观。实施城区环境综合整治，市区街路机械化清扫率达到85%，国家卫生城复审初步通过。千亩植物园对外开放，西湖湿地公园、南北河治理工程全面开工，南湖公园一期改造竣工开放。楼体风貌改造160栋，亮化提升206栋。出台规划监察管理办法，查处违章建筑17.1万平方米。我市成为国家智慧城市试点，智慧城管系统具备运行条件。新农村建设步伐不断加快，累计投入资金17.3亿元，集中打造12个精品片区，形成美丽乡村建设“四平模式”。生态环境明显改善。深入实施“四绿工程”，累计造林绿化104.7万亩。淘汰燃煤小锅炉、工业企业污染治理、餐饮业油烟净化、黄标车整治、秸秆禁烧等工作稳步实施。四平污水处理厂、梨树污水处理厂改造升级，全市出省境河流断面水质明显好转。

　　——全面深化改革加速推进。“放管服”改革成效初显。大力推进简政放权，实施并联审批，开展领办代办，服务效能明显提升。推进商事制度改革，实施“五证合一、一照一码”登记制度，推行“双随机、一公开”机制，联合惩戒失信市场主体，实现“一处违法、处处受限”。整合不动产登记职能，减少办证环节，减轻群众负担。经济领域改革率先突破。深入推行税制改革，“营改增”全面实施。出台金融产业发展实施意见，村镇银行实现县（市）区全覆盖。梨树县被确定为国家级农村金融综合改革试验区。制定管理规定，清理登记和规范管理国有资产。出台土地征收补偿安置办法，依法规范土地征收。四平成为全省唯一的地级供销社综合改革试点，经验在全省推广。教育文化等领域改革渐次展开。商业学校、轻化工学校等职业教育体制改革顺利推进。市艺术团、话剧团、电影公司转企改制圆满完成。安全生产监管防控、警务合成作战指挥系统建设等社会治理改革成效显著。

　　——人民生活水平持续改善。脱贫攻坚取得阶段性成果。2016年实施脱贫“五大工程”，推进“1+N”政策落实，投入资金7.3亿元，2.5万人实现脱贫，占全市贫困人口的37.1%。民生福祉显著提升。市本级财政收入77%以上用于民生事业，150件民生实事全面完成。全市累计新增就业18.8万人，农村劳动力转移就业215.7万人，城镇登记失业率控制在3.6%以内。机关事业单位养老保险改革全面启动，退休职工养老金实现12连增。城镇基本医疗保险待遇不断提高，临时救助制度全面建立，连续3年提高五保供养标准。累计改造棚户区238万平方米，改造暖房子710万平方米，百姓住得安全、住得温暖。城区二次供水改造工程使27万人受益，农村饮水安全工程让22.6万农村居民喝上安全洁净的放心水。新增、更新公交车229辆，其中新能源公交车40辆，群众出行更加便捷。社会事业全面进步。教育优先发展战略全面落实。医药卫生体制改革扎实推进，被评为“全国中医药先进单位”。图书馆、博物馆、大剧院等项目相继开工，文化“六馆”免费开放。体育馆、滑冰馆等项目建设进展顺利，我市被评为“全国群众体育先进单位”和“全国武术之乡”。民族团结、宗教和顺的良好局面持续巩固。国防动员和双拥共建深入开展，“全国双拥模范城”实现七连冠。“大国工匠”论坛成功举办，“四平工匠”“四平巧姐”受到表彰。妇女儿童、广播电视、知识产权、外事侨务、人民防空、地震监测、通信、气象、档案、老龄、地方志、残疾人等各项事业取得新成绩。社会大局和谐稳定。平安建设深入推进，24小时开展巡逻防控，两抢、盗窃和诈骗案件大幅下降，命案破案率达到100%。“六五”普法通过全省验收，法律援助体系覆盖城乡。安全生产形势稳定，持续开展隐患排查治理和专项整治，连续四年获得省政府目标责任制考核一等奖。食品药品安全“三大放心工程”启动实施，“四品一械”监管深入推进。加强应急管理，突发事件处置能力不断提升。信访形势明显好转，深入开展信访积案攻坚清零工程，一批信访积案得到有效解决，进京非访、去省集访量大幅下降，我市被评为“全省信访工作先进单位”。

　　——政府自身建设不断强化。自觉接受人大依法监督和政协民主监督，共办理人大代表建议360件、政协委员提案754件。启动政府立法工作，严格执行《四平市行政执法规定》，组建政府法律顾问团，荣获第四届“中国法治政府奖提名奖”。通过热线电话和门户网站倾听百姓呼声，一批群众关心的热点难点问题得到有效解决。制定出台《四平市行政问责办法》，建立政府重点工作任务落实管理系统，从严督导考核，刚性执纪问责，坚决整治政府系统不作为、乱作为等问题。深入开展党的群众路线教育实践活动、“三严三实”专题教育和“两学一做”学习教育，作风建设不断加强。

　　在看到成绩的同时，我们也清醒的认识到，全市经济社会发展中仍然存在一些亟待解决的突出问题：经济发展增速放缓，自主创新能力不足；稳增长促转型面临挑战，工业经济增长任务艰巨；资源环境承载压力不断增大，绿色发展任重道远；城市建管工作仍需强化，拆迁回迁等问题有待破解；财政收支矛盾比较突出，民生事业欠账较多；安全生产形势不容乐观，各种潜在风险依然存在；政府服务有待加强，发展软环境亟需改善。对这些问题，既要保持定力、坚定信心，又要做足准备，采取有效措施认真解决。

　　四年的奋斗历程，我们深刻体会到，做好新时期政府工作，必须坚持党的领导。切实增强“四个意识”，始终与中央和省委保持高度一致，坚决贯彻落实市委决策部署，牢牢把握正确前进方向。必须不断夯实基础。从最紧迫的事抓起，从最核心的事做起，把发展的基础筑得更牢，把振兴的根基夯得更实，一步一个脚印将事业推向前进。必须着力破解难题。始终坚持问题导向，顶住压力、保持定力，敢于面对困难，善于化解矛盾，在破解难题中迎难而上，在应对挑战中推动发展。必须勇于改革创新。坚决破除思维定式、工作惯性和路径依赖，向改革要动力、向创新要活力、向开放要空间，进一步激发振兴发展的强大力量。必须切实改善民生。始终坚持以人民为中心的发展思想，持续增加民生投入，提高公共服务能力，真正使发展成果更多更公平惠及全市人民。必须坚持依法行政。有效统筹协调各方利益，严格依法决策，自觉接受监督，切实按法定权限和程序履行职能。必须狠抓工作落实。坚持“干”字当先，“实”字为要，一届接着一届干，一张蓝图干到底，用实力、实绩、实效推进各项事业向前发展。

　　各位代表，过去四年取得的成绩，得益于市委科学决策、坚强领导，得益于市人大、市政协及社会各界有效监督、鼎力支持，得益于全市上下团结拼搏、共同奋斗。在此，我代表市政府，向全市人民，向人大代表、政协委员，向各民主党派、工商联、无党派人士和人民团体，向驻平人民解放军、武警官兵和政法干警，向中省直驻平各单位和关心支持四平发展的国内外朋友，表示崇高的敬意和衷心的感谢！

　　二、今后五年奋斗目标及2017年工作任务

　　今后五年，按照市七次党代会的决策部署，政府工作的总体要求是：高举中国特色社会主义伟大旗帜，坚持以马克思列宁主义、毛泽东思想、邓小平理论、“三个代表”重要思想和科学发展观为指导，全面贯彻十八大及十八届三中、四中、五中、六中全会精神，深入落实习近平总书记系列重要讲话精神，按照“五位一体”总体布局和“四个全面”战略布局，遵循“五大发展”理念，坚持稳中求进工作总基调，以深化供给侧结构性改革为主线，以提高发展质量和效益为中心，纵深推进“五路并进”“五市联动”“五城联创”，大力实施“五个率先”，全力构建“五大格局”，为打造新一轮东北振兴战略支点城市、建设“美丽四平、幸福家园”而努力奋斗！

　　今后五年，经济社会发展的奋斗目标是：全面完成“十三五”规划和市七次党代会提出的目标任务，经济增速和城乡居民收入增速均高于全省平均水平，经济结构、发展动能、城乡建设、民生福祉、治理能力实现升级，城市综合实力和整体竞争力大幅跃升。

　　——更加注重产业结构优化。三次产业结构不断优化升级，产业比重调整到19.6：40.2：40.2，率先在全省实现农业现代化，实现四平制造业创新转型，打造区域性服务业中心。高新技术产业产值所占比重逐步提高，民营经济主营业务收入大幅提升，商旅兴市取得更大成效。

　　——更加注重质量效益提升。一般公共预算财政收入占GDP比重进一步提高，发展平衡性、包容性、可持续性不断增强。发展空间格局进一步优化，开放型经济水平不断提升，加快打造“开放四平”。改革创新活力充分释放，“科技四平”建设取得突破，发展内生动力持续增强。

　　——更加注重民生福祉增进。脱贫攻坚任务全面完成。居民收入增长与经济发展保持同步。就业、社保、教育、医疗、住房等公共服务体系更加健全，基本公共服务均等化水平稳步提高。深入实施“五城联创”，城市基础设施日益完善，加快建设宜居宜业哈长城市群国家Ⅱ类大城市。

　　——更加注重生态环境改善。资源节约型和环境友好型社会建设取得重大进展，生产生活方式绿色、低碳水平不断提升，大气、水、土壤等污染得到有效整治。能源和水资源消耗、建设用地、碳排放总量和强度得到有效控制，主要污染物排放总量持续下降。

　　——更加注重文明程度提高。社会主义核心价值观更加深入人心，公民思想道德素质、文化素质、健康素质明显提高。全社会法治意识不断增强，“法治四平”建设取得重大进展。公共文化服务体系基本建成，文化影响力进一步提升。

　　——更加注重制度体系建设。重要领域和关键环节改革取得突破性进展，制约全市振兴发展的深层次矛盾和问题得到有效破解。民主政治建设持续增强，“平安四平”建设取得明显成效，社会更加和谐稳定，社会治理能力和水平显著提升。

　　2017年，是全面建成小康社会决胜阶段的关键之年，也是新一届政府的开局之年。全市经济社会发展的主要预期目标是：地区生产总值增长8%；工业增加值增长8.5%；服务业增加值增长10.7%；固定资产投资增长12%；一般公共预算地方级财政收入增长7%；社会消费品零售总额增长9.5%；外贸进出口总额增长7%；城乡居民人均可支配收入分别增长8%、7.5%。

　　今年我们将重点做好八个方面工作：

　　（一）保持发展定力，着力在稳增长中推动振兴。全力做强工业。稳定工业增长，加强运行调度，推动百威金士百销售结算中心落户我市，新增规模以上工业企业80户。推动转型升级，深化与建龙集团、中国建材集团等大企业合作，大力推进停产企业破产重整、升级改造，尽快使其恢复生产。积极筹建孟家岭建材产业园区。抓实工业提升，对接欧洲“工业4.0”，加快建设大数据、换热器、农机等千亿级产业群，争创“中国制造2025”试点示范城市，工业技改投资增长10%，首次投产新产品达到120种以上。大力发展民营经济，深入落实扶持政策，全年助保贷款规模达到2亿元，新登记民营企业2800家、个体工商户1.6万户，民营经济主营业务收入增长11%。攻坚重点项目。计划建设5000万元以上项目150个,春季集中开工项目必保达到30个以上。推进世宝汽车铸件智能化改造等60个重点项目开工建设,大清鹿苑鹿产业园等90个在建项目全面复工，上半年确保线路器材厂转型改造等10个项目竣工投产。对重点项目实行清单式管理，进行月调度、季总结，推动项目审批由上报式向下沉式转变，由互为要件向并联审批转变，由企业办理向领办代办转变，做到落地项目快开工、开工项目快投产、投产项目快见效。狠抓招商引资。立足我市区位、产业、资源等优势，主动融入“一带一路”建设，瞄准环渤海、长三角、珠三角等热点地区，围绕装备制造、农产品加工、现代物流、新型建材等重点产业，吃透内情、掌握外情，知己知彼、主动出击，通过采取小分队专业化定向性的方式，进一步提升招商工作的精准度和实效性。深入对接世界500强、大型央企等战略投资者，推动“四平－辽源－铁岭－通辽”经济协作区发展，建立发展国内外友好城市，提升对外开放水平。加快园区建设进度，做强载体平台，发挥龙头作用，优化产业布局。强化要素供给。增强资金支撑，健全完善政府和社会资本合作机制，多领域推广PPP模式。优化政府投资方向，充分发挥引导带动作用。全年向上争取专项资金增长15%。提升金融服务，降低融资成本，扩大再担保机构覆盖范围。加快企业上市步伐，推进实现A股上市。优化政策资源配置方式，建立重大项目用地报批联动机制，集中优质要素向大项目、好项目倾斜。

　　（二）强化创新引领，着力在促升级中提质增效。坚持创新驱动发展战略。做好“无中生有”“有中生新”两篇文章，进一步增强发展内生动力。举办第二届中国·四平科技成果展洽会，继续开展“院士专家四平行”等活动，实施“四平创造”工程，推进域内企业与科研院所、高等院校深度合作，引导更多创新要素汇聚四平。加强科技大市场后续建设，加快四平换热器国检中心和省换热设备技术研发中心改革步伐，提升换热器、农机等设备装备公共技术研发平台建设水平。依托四平经济开发区谋划建设省级高新技术开发区，建立智能制造、现代农机、专用汽车等6个院士工作站。深化供给侧结构性改革。扎实推进“三去一降一补”重点任务，通过化解过剩产能甩掉包袱、轻装上阵。加大棚户区改造货币化安置力度，引导房地产市场健康有序发展；拓宽粮食销售渠道，确保农民种粮卖得出。支持企业市场化、法治化债转股，加大股权融资力度，降低企业杠杆率。优化信贷投向，提高直接融资比重，有序推进政府存量债务置换。着力抓好降成本措施落地见效，按照国家和省里政策适当减免企业税费。开展质量提升行动，发扬工匠精神，强化品牌建设，提高企业产品质量和核心竞争力。加快产业结构调整步伐。围绕“三四三”产业体系，着力推动传统产业与科技嫁接、与设计联姻、与品牌联动，打造“中国换热器城”“中国农机城”。大力发展光电、医药健康、新能源汽车等新兴产业，推进吉高物流等现代物流项目建设。深化现代服务业跃升攻坚，完成金融街工程主体封闭，推动万嘉购物广场、欧亚商业综合体等重点项目破土动工，加快农村电商发展，筹建网上四平特色商品馆，全年新增商业限上企业20户。加快推进“旅游城”建设，精心打造二龙湖国际旅游度假区，规划建设塔子山红色旅游景区，推进一江山岛生态公园、叶赫满族旅游区等重点旅游项目建设，打造国家旅游装备制造业集聚区，全面发展红色旅游、满族文化旅游、休闲度假旅游和冰雪娱乐旅游。

　　（三）发展现代农业，着力在强基础中激发活力。推动农村改革创新。积极推进“三权分置”，全面完成土地确权登记颁证工作。扎实抓好第三次全国农业普查。深化农村金融、林业、供销社等领域改革创新，扎实推进“农地贷”，探索建立粮食收购贷款信用保证基金，拓宽农村融资渠道。推进农业转型升级，优化农产品供给结构，提高农产品供给质量。加快“三大体系”建设。构建现代农业产业体系。加快玉米产业化发展，推进玉米精深加工，重点抓好天成玉米年产13万吨氨基酸和5万吨L—乳酸等农业产业化项目。构建现代农业生产体系。调整粮食种植面积100万亩，新建扩建棚膜园区15个、标准化牧业小区100个。加强黑土地保护治理，扎实推进万亩现代农业高标准示范农田建设，加快河湖连通、农田水利、农业节水灌溉等工程建设。落实农机购置补贴政策，耕种收全程机械化水平达到85%。构建现代农业经营体系。着力培育专业大户、农民合作社等新型农业经营主体，引导合作社开展内部信用合作，农村适度规模经营比重达到50%以上。强化农产品品牌建设，新增“三品一标”认证农产品100个。突出发展县域经济。做大经济总量，提升发展质量，努力走出一条城乡统筹、产城融合、各具特色的县域发展之路。推进县域优势产业集群发展，提高产业发展层次和水平。发展特色城镇，打造一批具有浓郁本土特色的工业强镇、农业大镇、商贸重镇、文化名镇和旅游旺镇。提升美丽乡村品质。推进省级重点项目建设，集中打造提升15条基础条件好、带动能力强、提升空间大的精品线路。加快农村“五改”步伐，争创省级美丽乡村10个，评选美丽庭院5000个、干净人家2万户。

　　（四）建设宜居城市，着力在补短板中实现赶超。加快基础设施建设。着眼打造哈长城市群国家Ⅱ类大城市，启动城市总体规划修编、中心城区控制性详细规划、城市交通规划、水资源综合规划、新型工业化经济开发区及二龙湖生态保护示范区等规划编制。续建管廊14条、新建管廊11条，形成廊体25公里。全力推进海绵城市建设，争取成为国家试点。新建城区道路18条，新增道路里程12公里。扎实推进康平街、慧智街公铁立交桥建设，紫气大路、东丰路公铁立交桥完成转体合拢。重点实施绥沈公路、牙四公路、二龙湖至叶赫旅游公路项目建设，加快推进四平至伊通一级公路项目，配合省交通厅全面完成集双高速四平境内征地拆迁任务。新建天然气门站1座，改造老旧燃气管网20公里。积极推进引松入平工程，全面实施污水处理厂二期改造、北河截流干管等工程。提升城市管理水平。推进城市管理精细化，加大规划监察力度，依法依规拆除城市各类违建，巩固国家卫生城创建成果。推进城市管理制度化，完善城市管理体制和机制，打造市区纵向联动、部门横向呼应的执法模式。加快建设智慧城市，扎实推动全域大数据中心云平台、公共基础数据库、智慧管网子系统等项目建设。持续优化生态环境。加强大气污染防治和监管，实施机动车和非道路移动源污染控制工程，彻底淘汰在用黄标车，大力推进秸秆产业发展。加快污水处理及配套管网建设，加大重点河流污染综合整治力度，集中式饮用水源地水质稳定达标。造林30万亩，绿化美化村屯354个。加快架树台湖国家湿地公园建设。谋划东辽河干流综合治理等重大水利项目，加快推进南北河城市防洪工程、中小河流治理、东山动植物园、西湖湿地公园、南湖公园二期等重点建设工程。

　　（五）全面深化改革，着力在破难题中增强动力。加快推进“放管服”改革，完善权力清单、责任清单，公开发布行政审批事项目录，建立健全监督检查机制。成立专家咨询团，提高政府决策的科学化民主化水平。整合政府采购、建设项目招投标、土地使用权和矿业权出让、国有产权交易等交易服务职能，建立统一的公共资源交易平台，提高公共资源配置效率和效益。深入推进商事制度改革，巩固“先照后证”改革，抓好企业“五证合一”和个体工商户“两证整合”向“多证合一”改革转变；全面推行“双随机、一公开”监管制度，实施失信企业协同监管和联合惩戒，努力打造诚信环境。加快重点领域突破，积极推进财税改革，重点做好市对区（开发区）财政体制调整工作。全面推进综合治税，建立综合治税大数据网络平台。鼓励发展银行业金融机构，谋划建立工业、农业、服务业、城建发展基金。全面推进水务体制改革，逐步实现原水、引水、净水、供水、排水、污水处理及中水回用的一体化管理和市场化运营。组建四平职教集团，整合涉农职业教育资源，打造四平特色职教品牌；深入推进办学体制及考试制度改革。深化医药卫生体制改革，加快分级诊疗制度建设，启动公立医院综合改革。深化律师制度改革，有效发挥公职律师、公司律师和社会律师服务职能。全面推进农垦改革，努力破解辽河垦区体制机制问题。

　　（六）改进社会治理，着力在保稳定中促进和谐。深化平安建设。加强社会综合治理，整合现有资源，建立联动机制，提升社会治理效率。全面构建点线面结合、网上网下结合、人防物防技防结合、打防管控结合的立体化社会治安防控体系，实现平安建设全覆盖。夯实公共安全管理，推行对重点车辆、易发事故重点路段的常态监管机制。加强消防安全管理，做实做精“微型消防站”。开展法治宣传教育，深入推进“七五”普法工作。全面贯彻落实党和国家民族宗教政策。抓实信访维稳。严格执行《四平市信访工作责任追究办法》，层层传导压力，推动信访工作重心下移、关口前移。加强源头预防，实行重大项目重大决策社会稳定风险评估。打好信访积案化解“清零”攻坚战，对缠访闹访、以访施压、以访谋利及组织串联进行大规模聚集的上访人员依法打击，以法治力量驱动信访工作水平全面提升。强化安全生产。坚持“党政同责、一岗双责、齐抓共管、失职追责”安全生产责任制，突出抓好企业主体责任落实，深入开展安全风险分级管控和隐患排查治理双重预防性机制建设，坚决遏制重特大事故发生。完善企业安全生产不良记录“黑名单”制度，强化安全基础保障和应急救援能力。持续推进食品药品安全放心工程，全力构建市县乡村四级监管体系，努力创建国家食品安全示范城市。

　　（七）坚持执政为民，着力在惠民生中共享发展。推动创业就业。深入推进大众创业、万众创新，强化创业创新平台建设，实施大学生创业引领、就业促进、帮扶援助“三项计划”，激发创业创新热情。深入开展“就业援助月”和“春风行动”等活动，城镇新增就业4万人，城镇登记失业率控制在4.5%以内。提升社会保障水平。坚持以基本养老、基本医疗、最低生活保障为重点，加大财政投入力度，提升社会保障水平。继续推进机关事业单位工作人员养老保险制度改革，确保制度并轨平稳有序。实施养老服务普惠推动计划，规范完善社区居家养老服务功能，逐步扩大政府购买居家养老服务范围。加大社会救助工作力度，深入开展“捐资助学”“助医助困”等慈善救助活动。大力发展社会事业。实施文化惠民工程，扎实开展文化“七进”，加快推进图书馆、博物馆、大剧院等项目建设。确保滑冰馆项目开工、体育馆全面竣工。筹备举办第22届全国百城市自行车赛四平赛区决赛。深入实施学前教育“三年行动计划”，推进义务教育优质均衡发展，促进普通高中教育特色化，加强民族教育和特殊教育，提升教育信息化建设水平，推动各阶段教育教学质量全面提升。围绕打造国家级“健康城”，加快医疗卫生事业发展，加强计划生育工作，突出发展中医药事业，推动疾控中心、结核病防治所、城市社区卫生服务中心等项目加速建设。进一步实施精准扶贫。聚焦“两确保、两不愁、三保障”目标，扶贫先扶志，“输血”更“造血”，强化特色产业发展、基础设施建设、公共服务保障和资金管理使用，确保完成2017年脱贫攻坚目标。在全面做好各项民生工作基础上，重点完成棚户区改造、市政道路建设等35件民生实事，持续提升人民群众幸福感、满意度。

　　（八）加强政府建设，着力在抓落实中担当作为。新形势新任务对政府工作提出了新要求，我们必须以新理念引领政府工作创新突破。大力解放思想，做到敢创新、求突破。牢固树立“一二三”意识，破除求稳守成观念，鼓励大胆地试、大胆地闯；破除坐而论道、知行不一的不良风气，进一步把解放思想落实到解决问题上、落实到工作成效上。坚持求真务实，做到讲实话、干实事。立足于“实”、扎根于“实”、落脚于“实”，以敬事为民的崇高追求，百折不挠的奋斗精神，集中精力解决问题，凝心聚力推动发展，精准发力攻坚克难。勇于大胆干事，做到有担当、有作为。坚决从墨守成规中走出来，从按部就班中走出来，以“朝受命、夕饮冰”的紧迫感和“昼无为、夜难寐”的责任感，担重任、挑大梁，迎难而上、披荆斩棘、勇往直前。推进依法行政，做到重法治、严规范。自觉接受人大法律监督、政协民主监督以及社会监督。继续推进政府立法工作，加大政府信息公开力度，健全依法决策机制，规范公正文明执法，切实加强法治政府建设。强化激励问责，做到想干事、干成事。建立健全容错纠错、正向激励和整改问责的长效机制，鼓励大胆干事、竞进作为。严格执行《四平市行政问责办法》，坚决克服懒政、怠政，坚决惩处失职、渎职，着力营造真抓实干、狠抓落实的浓厚氛围。加强廉政建设，做到自身净、守规矩。巩固深化党的群众路线教育实践活动、“三严三实”专题教育和“两学一做”学习教育成果，持之以恒纠正“四风”。严肃党内政治生活，加强党内监督，严格落实全面从严治党政治责任，加强行政监察和审计监督，认真落实问题整改，严查各类腐败案件，全力推动廉洁政府建设。

　　各位代表，宏伟蓝图令人鼓舞，美好愿景催人奋进。让我们紧密团结在以习近平同志为核心的党中央周围，在省委、省政府和市委的坚强领导下，改革创新，攻坚克难，扎实工作，为全力打造新一轮东北振兴战略支点城市，加快建设“美丽四平、幸福家园”而努力奋斗！
